# Multiple sclerosis and gut microbiota: Lachnospiraceae from the ileum of MS twins trigger MS-like disease in germfree transgenic mice—An unbiased functional study

**DOI:** 10.1073/pnas.2419689122

**Published:** 2025-04-21

**Authors:** Hongsup Yoon, Lisa Ann Gerdes, Florian Beigel, Yihui Sun, Janine Kövilein, Jiancheng Wang, Tanja Kuhlmann, Andrea Flierl-Hecht, Dirk Haller, Reinhard Hohlfeld, Sergio E. Baranzini, Hartmut Wekerle, Anneli Peters

**Affiliations:** ^a^Institute of Clinical Neuroimmunology, University Hospital Ludwig-Maximilians-Universität München, Martinsried 82152, Germany; ^b^Biomedical Center, Faculty of Medicine, Ludwig-Maximilians-Universität München, Martinsried 82152, Germany; ^c^Max Planck Institute for Biological Intelligence, Martinsried 82152, Germany; ^d^Munich Cluster of Systems Neurology, Munich 81377, Germany; ^e^Department of Medicine II, University Hospital, Ludwig-Maximilians-Universität München, Munich 81377, Germany; ^f^Weill Institute for Neurosciences, Department of Neurology, University of California San Francisco, San Francisco, CA 94158; ^g^Institute of Neuropathology, University Hospital Münster, Münster 48153, Germany; ^h^Zentralinstitut für Ernährungs- und Lebensmittelforschung Institute for Food and Health, Technical University of Munich, Freising 85354, Germany

**Keywords:** CNS autoimmunity, MS/EAE, microbiota, gut–brain axis, gnotobiotic mice

## Abstract

We developed a two-tiered strategy aiming to identify gut bacteria functionally linked to the development of multiple sclerosis (MS). First, we compared gut microbial profiles in a cohort of 81 monozygotic twins discordant for MS. This approach allowed to minimize confounding effects by genetic and early environmental factors and identified over 50 differently abundant taxa with the majority of increased taxa within the *Firmicutes*. These included taxa previously described to be associated with MS (*Anaerotruncus colihominis* and *Eisenbergiella tayi*), along with newly identified taxa, such as *Copromonas* and *Acutalibacter*. Second, we interrogated the intestinal habitat and functional impact of individual taxa on the development of MS-like disease. In an exploratory approach, we enteroscopically sampled microbiota from different gut segments of selected twin pairs and compared their compositional profiles. To assess their functional potential, samples were orally transferred into germfree transgenic mice prone to develop spontaneous MS-like experimental autoimmune encephalomyelitis (EAE) upon bacterial colonization. We found that MS-derived ileal microbiota induced EAE at substantially higher rates than analogous material from healthy twin donors. Furthermore, female mice were more susceptible to disease development than males. The likely active organisms were identified as *Eisenbergiella tayi* and *Lachnoclostridium,* members of the Lachnospiraceae family. Our results identify potentially disease-facilitating bacteria sampled from the ileum of MS affected twins. The experimental strategy may pave the way to functionally understand the role of gut microbiota in initiation of MS.

## Significance

We developed a strategy to identify gut bacteria functionally linked to the development of multiple sclerosis (MS). To minimize confounders, we analyzed microbiota composition in a large cohort of monozygotic twins discordant for MS and identified over 50 differently abundant taxa. We then sampled microbiota from the ileum of selected twins, and, in order to functionally characterize them, we introduced them into germfree TCR-transgenic mice prone to develop MS-like disease upon bacterial colonization. We found that MS-derived ileal microbiota induced disease at higher rates than analogous material from healthy twin donors. Our results implicate two Lachnospiraceae members, namely *Eisenbergiella tayi* and *Lachnoclostridium,* as likely responsible for an increased incidence of disease.

Multiple sclerosis (MS), the most prevalent inflammatory demyelinating disease of the central nervous system (CNS), develops in people with genetic susceptibility likely after encountering a yet-unknown roster of environmental factors. The risk factors comprise more than 200 common DNA variants and a broad range of diverse extrinsic stimuli, including smoking, deprivation of vitamin D, obesity, and infection with Epstein–Barr virus (EBV) (1). Furthermore, an unexpected class of microbial organisms, commensal gut microbes, have gained attention as possible disease-precipitating agents (2). Large population-based studies revealed that MS is accompanied by changes of the intestinal microbial composition (3). However, the changes reported by different investigators were often discordant, if not contradictory between studies. Some of this discrepancy may be explained by the genetic diversity of individuals within and between cohorts, as well as by inhabiting different regions and exhibiting nonuniform nutritional habits (3–6). In addition, with few exceptions (7–9), these investigations remained largely descriptive, providing no mechanistic insights into the function of MS-related microbial profiles.

Compelling functional evidence can be obtained from the transfer of fecal samples into germfree (GF) transgenic mice with genetic disposition to develop MS-like CNS inflammation, namely experimental autoimmune encephalomyelitis (EAE). Transgenic mice of the RR strain express a receptor recognizing the autoantigen myelin oligodendrocyte glycoprotein (MOG) on most of their T cells and develop an inflammatory demyelinating CNS disease that recapitulates the early stage of human MS. In this EAE model, disease develops spontaneously, as long as the animals are raised in specific-pathogen-free (SPF) housing conditions. However, when RR mice are raised in GF conditions, they remain almost completely healthy. Colonization of the GF transgenics with microbiota from healthy SPF-bred mice led to the development of EAE (10), suggesting that intestinal microbiota have disease-triggering potential. EAE was also induced by human fecal microbiota and importantly, fecal samples from people with MS were more efficient in triggering disease than material from healthy donors (7).

Our previous investigations indicated a functional relationship between MS-derived intestinal microbiota and the initiation of CNS autoimmune disease, but did not identify the nature of disease-related bacteria, nor their location within the intestinal tract. Here, we developed a unique experimental strategy by studying fecal microbiota on a large cohort of monozygotic twins discordant for MS, an approach that drastically reduces potential confounding factors that could bias results. In an exploratory approach, we then sampled microbiota from small and large intestine from selected twins and introduced those human-derived bacterial samples into GF transgenic mice. Our results serve as a proof of concept that human ileal microbiota harbor pathogenic disease-triggering species and implicate two members of the Lachnospiraceae family as potentially responsible for an increased incidence of clinical EAE.

## Results

### The Fecal Microbiome of MS-Discordant Monozygotic Twins.

**Fig. 1.**
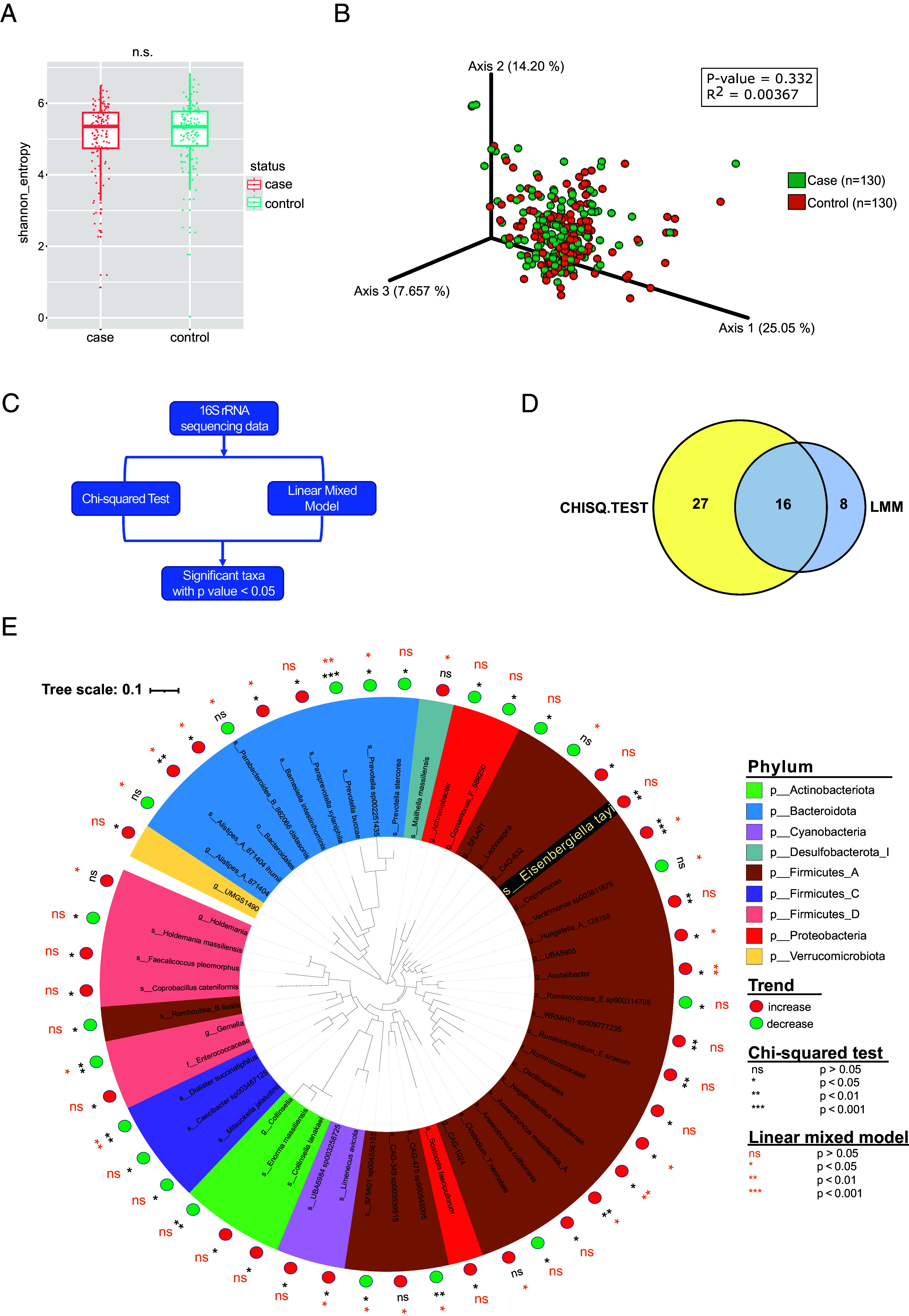
16S rRNA sequencing analysis of fecal microbiota from 81 twin pairs. (*A*) Alpha diversity (computed as Shannon entropy) was not significant between affected and unaffected twins (Mann–Whitney *U* test; *P* = 0.7989). (*B*) Beta diversity (PERMANOVA test) across affected and unaffected twins was not significant (*P* > 0.3; r^2^ = 0.004). (*C*) Two statistical tests were performed on the data (Linear mixed model and Chi-squared). Taxa nominally significant in any test were evaluated further. (*D*) Venn diagram showing the nominally significant taxa for each test (24 taxa for LMM and 43 taxa for Chi-squared). A total of 51 taxa were significant in at least one test, while 16 taxa were significant in both. (*E*) A phylogenetic tree showing the 51 taxa nominally significant in at least one test. The “Trend” in the legend indicates changes in abundance in affected twins (compared to unaffected twins). Font color corresponds to the significance of the corresponding statistical test (black: Chi-squared; orange: LMM).

The Munich MS Twin Study currently assembles 101 monozygotic twin pairs clinically discordant for MS (11, 12). We collected fecal samples from 81 pairs (at least one time point; for some pairs up to three time points) and analyzed microbial profiles via 16S rRNA amplicon sequencing. As expected, and in line with previous studies, no significant differences were found in either alpha or beta diversity (Fig. 1 *A* and *B*). To take full advantage of the monozygotic twin study design, we implemented a linear mixed model (LMM) with a fixed effect of disease status, and a random effect of twinship as a measure to account for differences between the discordant twins and reduce the effect of additional confounding variables. Acknowledging the limited statistical power of our sample size, we also performed Chi-squared analysis to identify taxa that were either increased or decreased in the affected twins (Fig. 1*C*). Out of all identified taxa (*SI Appendix*, Fig. S1), a total of 51 taxa were identified as differentially abundant in either test (27 by Chi-squared, 8 by LMM), and 16 of them were identified by both (Fig. 1*D*). Fig. 1*E* shows the phylogenetic tree of all 51 taxa, colored by Phylum. Several taxa, including the propionate producers *Dialister succinatiphilus* and *Prevotella buccae*, were found to be significantly decreased in MS twins by both statistical tests (green dots in Fig. 1*E*). Furthermore, *Alistipes ihumii*, a species unable to convert tryptophan to indol (13), was significantly increased in both tests (Chi-squared, *P* < 0.001, LMM *P* < 0.05). This is of particular interest, as indol is one of the tryptophan-derived metabolites that has been associated with immunomodulatory properties (14–16). In addition, significantly increased taxa were enriched within the *Firmicutes,* including, for example, *Copromonas*, *Acutalibacter*, and *Anaerotruncus massiliensis* (red dots in Fig. 1*E*). While 16S rRNA sequencing cannot fully resolve at the species level in all cases, *Eisenbergiella tayi* was identified as one of the organisms significantly increased in MS twins (Chi-squared, *P* < 0.01). This finding is of interest in relationship with the subsequent analysis.

### Site-Specific Microbial Signatures in MS-Discordant Twin Intestines.

Our approach to juxtapose fecal microbial profiles of identical twins with and without MS uncovered multiple disease-associated changes in abundance of various microbial taxa. However, these associative alterations per se fail to identify those taxa responsible for triggering clinical disease. We designed a strategy to fill this gap, by first sampling microbes from the ileum, the intestinal site that most probably harbors the highest frequencies of proinflammatory Th17 cells in mice, which we hypothesize to be essential for disease triggering. As a second element of the strategy, we introduced this material into germfree transgenic mice that portray the early stage of human MS dependent on microbial colonization.

In order to explore microbial composition at different intestinal sites, we selected four pairs from the twin study who volunteered to undergo enteroscopy. This subcohort is disparate in gender (3/4 female), clinical course (3 RRMS and 1 SPMS), disability (EDSS 1 to 6), age (35 to 58 y), duration of disease (1 to 23 y), and treatment (*SI Appendix*, Table S1). Importantly, all participants lived in their parental home until about age 20, and twins largely shared lifestyle factors such as dietary and smoking habits. After routine bowel cleansing procedures, we sampled microbiota from the terminal ileum, and colon by enteroscopy. From each site, luminal fluid was aspirated and mucosal biopsies were taken to map luminal and mucosa-associated microbiota in comparison with fecal microbiota. We profiled the microbiota via 16S rRNA amplicon sequencing and performed pairwise comparisons. Overall, alpha diversity (measured by richness and Shannon effective) of the different intestinal sites was largely indistinguishable between the MS and healthy siblings (*SI Appendix*, Fig. S2*A*). Further, neither the ileal and colonic nor the fecal compartment showed significant differences in beta diversity between healthy and MS twins (*SI Appendix*, Fig. S2*B*).

**Fig. 2.**
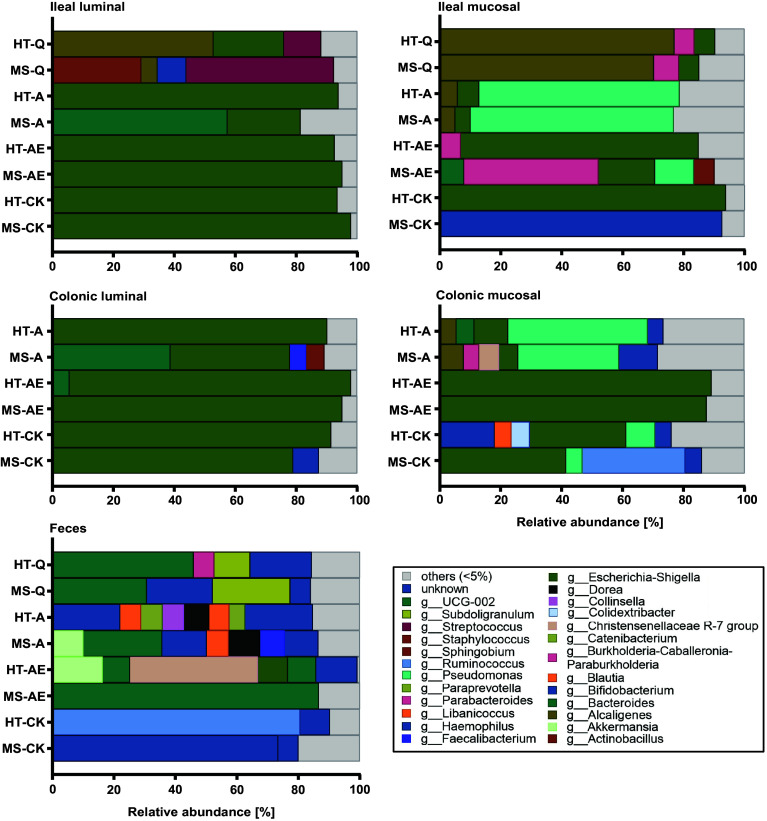
16S rRNA sequencing analysis of enteroscopically obtained microbiota from different intestinal sites. Microbiota from the lumen and mucosa of the terminal ileum and the colon were sampled during ileocolonoscopy in four volunteering twin pairs and analyzed together with fecal samples by 16S rRNA amplicon sequencing. Graphs show relative abundance at the genus level of the location-specific intestinal microbiota. Genera with a relative abundance of <5% were grouped as “others.”

However, some discrepancies between the profiles within the discordant twin pairs appeared at the genus level. Fig. 2 shows the paired taxonomic profiles from the four different intestinal sites (ileal lumen, ileal mucosa, colonic lumen, and colonic mucosa), as well as from feces from all four twin pairs. As expected, the composition of luminal microbiota contrasted strongly from mucosa-associated microbiota, and all of the enteroscopically obtained profiles differed substantially from those in the fecal samples (Fig. 2 and *SI Appendix*, Fig. S2*C*). Some genera, such as *Escherichia/Shigella*, were highly abundant in enteroscopically obtained luminal microbiota but were barely represented in feces. Notably, the similarity of microbiota composition *within* each twin pair was considerably higher than between unrelated people with the same disease status (Fig. 2 and *SI Appendix*, Fig. S2*C*).

### Colonization with MS-Derived Ileal Microbiota Triggers EAE.

We previously reported (7) that colonization of asymptomatic GF RR mice with fecal microbiota from human donors facilitated development of spontaneous EAE, and fecal microbial samples from MS-afflicted donors were more efficient at triggering disease development than samples from the healthy donors. We now refined our experimental strategy to determine the location of the disease-facilitating bacteria in the human intestine and to identify the active organisms.

For the first colonization study, we selected one female MS twin (MS-Q), showing a particularly discrepant ileal profile to her unaffected twin (HT-Q) (Fig. 2). MS-Q presented with a disease duration of 23 y with initially relapsing–remitting disease course but coursing with SPMS and an EDSS of 6.0 at the time of this study. Importantly, at the time of enteroscopy, the MS twin had not been using disease-modifying treatment for more than 10 y (*SI Appendix*, Table S1).

**Fig. 3.**
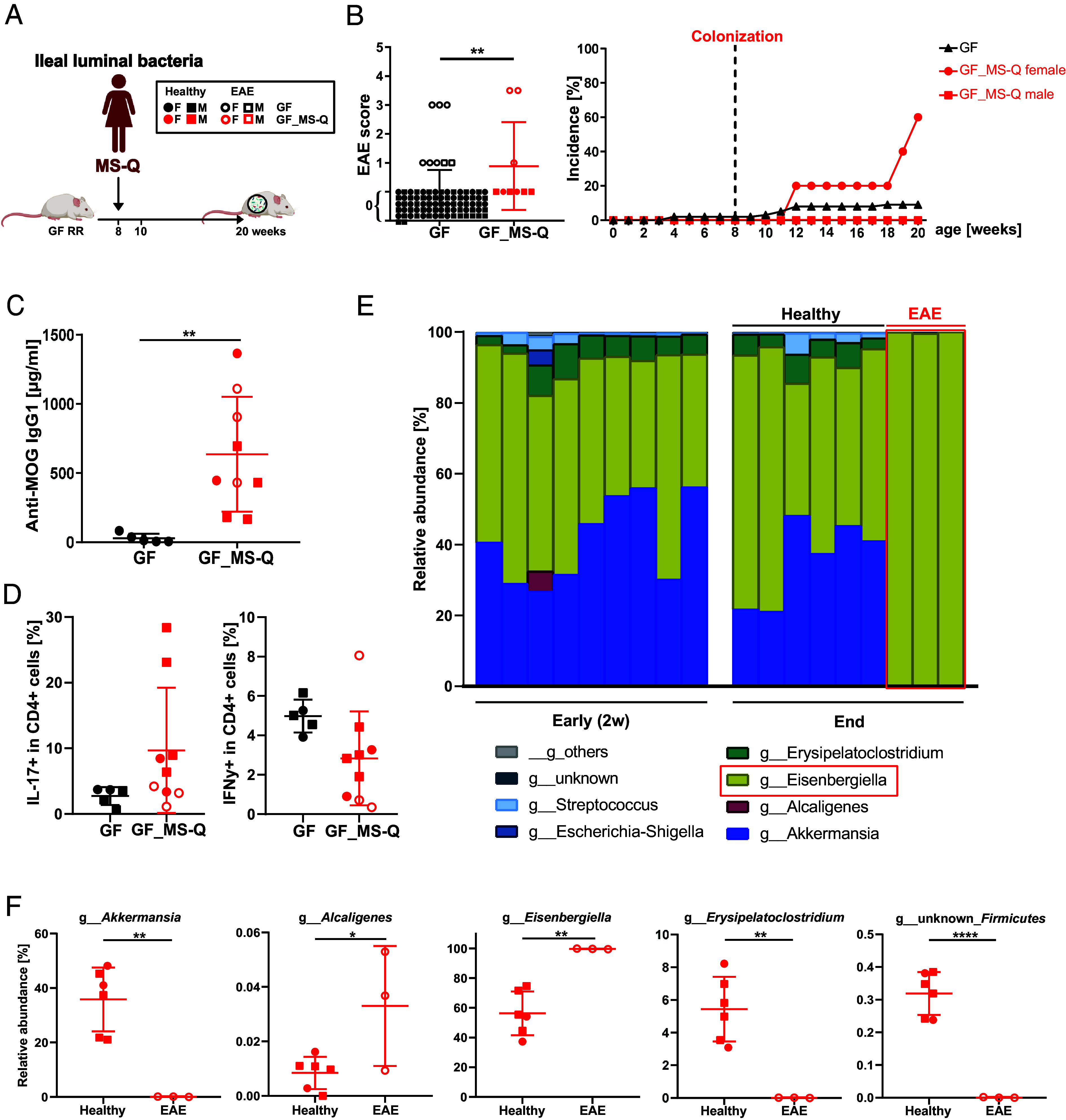
Colonization with ileal MS twin–derived microbiota can trigger CNS inflammation. (*A*) Experimental scheme: Gnotobiotic RR SJL/J mice were gavaged with ileal bacteria from an MS patient (MS-Q, n = 9). Created with BioRender. (*B*) EAE scores and incidence of spontaneous EAE in humanized gnotobiotic RR mice compared to the entire GF colony (n = 85) (unpaired *t*-test). (*C*) Anti-MOG IgG1 was measured by ELISA in the serum of humanized gnotobiotic RR mice compared to GF RR mice (unpaired *t* test). (*D*) Fractions of IFNγ- and IL-17-producing CD4^+^ T cells in the spleen of humanized gnotobiotic RR mice were assessed by flow cytometry. (*E*) Relative abundance of different genera of the fecal microbiota from humanized gnotobiotic RR mice 2 wk after colonization (early) and at the endpoint. *Eisenbergiella*, the dominant genus in sick RR mice at the endpoint, is framed in red. (*F*) Relative abundance of fecal microbiota in recipients of MS patient-derived ileal microbiota that showed a significant difference (unpaired *t*-test) between healthy and diseased animals.

Inspired by previous reports, which linked the ileal-associated immune tissues to autoimmune processes (17, 18), we started out by transferring samples from the terminal ileum of MS-Q into GF RR mice (Fig. 3*A*). Indeed, within 4 to 12 wk after colonization, spontaneous EAE arose in 3/9 recipient mice (Fig. 3*B*). Importantly, all affected animals were female (3/5) (Fig. 3*B*). The disease presented all clinical and histological features of classical EAE (19), including paresis of tail and hind limbs, cellular infiltrates in the spinal cord and confluent fields of demyelination (*SI Appendix*, Fig. S3*A*). All mice showed similar colonization density ranging between 2 to 13 × 10^11^ bacteria/g of feces (*SI Appendix*, Fig. S3*B*). Irrespective of disease status, serum anti-MOG IgG1 antibody titers rose significantly in colonized RR mice compared to the GF colony (Fig. 3*C*). In addition, the fraction of IL-17-producing CD4^+^ T cells showed a trend to be increased in the spleen, whereas the fraction of IFNγ^+^ CD4^+^ T cells trended toward a decrease compared to the GF RR mice (Fig. 3*D*). Interestingly, microbial colonization was associated with formation of lymphocytic infiltrates in both cecum and proximal colon of colonized mice compared to GF mice (*SI Appendix*, Fig. S3*C*), suggestive of mild intestinal inflammation.

We profiled the fecal microbiota of all gnotobiotic mice 2 wk after colonization and the fecal and ileal microbiota at the endpoint by 16S rRNA gene amplicon sequencing. Examination of the recipients’ microbial profiles showed a tight association between microbial composition and the presence of EAE. Remarkably, all sick mice displayed dominance of one particular species in both feces (Fig. 3*E*) and ileum (*SI Appendix*, Fig. S3*F*), which was classified as *Eisenbergiella tayi*, a member of the Lachnospiraceae family. This dominance was reflected in a reduction in alpha diversity in sick mice and distinct microbial profiles of sick *versus* healthy mice, as shown by beta diversity (*SI Appendix*, Fig. S3 *D* and *E*). In all 3 sick mice, the dominance of *E. tayi* appeared at the endpoint of the experiment but was less pronounced in earlier fecal samplings preceding the onset of disease (Fig. 3*E*). This came at the expense of other bacterial genera such as *Akkermansia* and *Erysipelatoclostridium*, both of which were substantial components in all recipient mice at the early time point. However, this pattern remained stable through the end of the experiment only in healthy mice but was no longer detected in EAE mice (Fig. 3*F*). Of interest, *E. tayi* was also found increased in the feces of most MS twins (Fig. 1*E*).

### Colonization with Ileal Microbiota from MS Versus Healthy Twins.

**Fig. 4.**
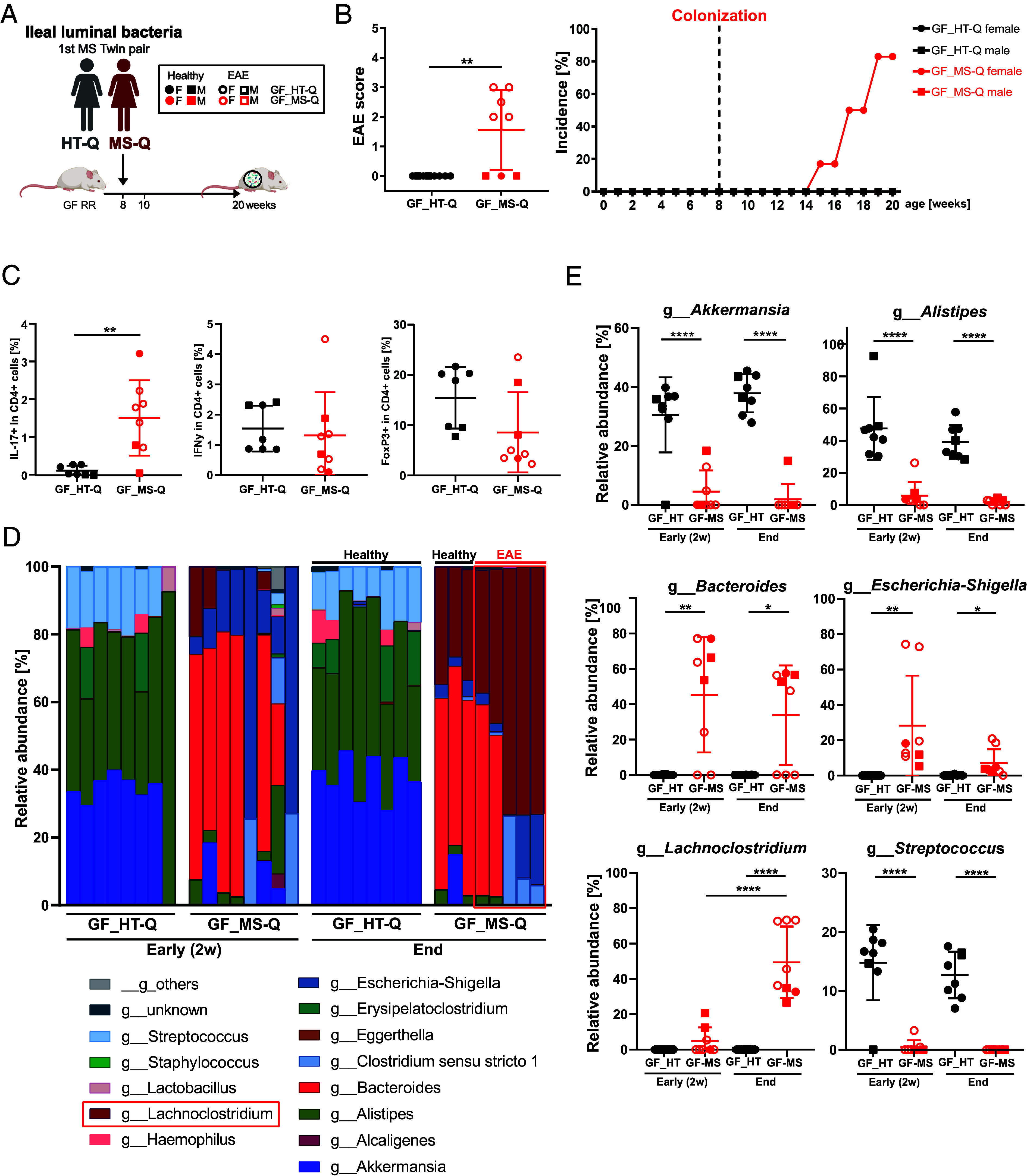
Ileal microbiota play a pivotal role in triggering CNS inflammation. (*A*) Experimental scheme: Gnotobiotic RR SJL/J mice were colonized with ileal bacteria from the MS twin (GF_MS-Q, n = 8) or healthy cotwin (GF_HT-Q, n = 8). Created with BioRender. (*B*) EAE scores and incidence in colonized mice (unpaired *t*-test). (*C*) The percentage of IFNγ- and IL-17-producing CD4 T cells and FoxP3+ Treg cells in the spleen of colonized RR mice (unpaired *t*-test). (*D*) Relative abundance of different genera of the fecal microbiota from colonized RR mice 2 wk after colonization (early) and at the endpoint. *Lachnoclostridium* was enriched in MS-Q colonized RR mice at the endpoint and is framed in red. (*E*) Relative abundance of fecal microbiota from colonized RR mice that showed significant changes (unpaired *t*-test) at the genus level.

In an independent transfer experiment, we directly compared the EAE-inducing potential of ileal material from MS-Q with the unaffected cotwin HT-Q. Of note, these twins lived in the same household until age 20 and thus share not only genetic but also early environmental factors. We colonized 8 GF RR mice (2 males and 6 females) with ileal material from MS-Q and another 8 RR mice (2 males and 6 females) with ileal samples from HT-Q (Fig. 4*A*). Similar to our preceding experiment, colonization with MS-derived ileal microbiota was followed by EAE development in 5 out of 6 female and 0 out of 2 male RR recipients after 7 to 12 wk. In contrast, all recipients of ileal samples from HT-Q remained healthy (Fig. 4*B*). Inflammatory and demyelinating lesions presented only in the CNS of recipients colonized with ileal material from MS-Q but not in that from HT-Q (*SI Appendix*, Fig. S4*A*). The infiltrates contained modest amounts of T cells and few B cells but were dominated by activated macrophages (*SI Appendix*, Fig. S4*A*). MOG-specific IgG1 antibody titers were indistinguishable in recipients of samples from MS-Q and HT-Q (*SI Appendix*, Fig. S4*B*).

As in the initial experiment, analysis of the CD4 T cell response showed a significant increase in Th17 cells in the spleen, whereas Foxp3^+^ Tregs trended to be decreased in the spleen of recipients of MS-Q ileal material compared to recipients of ileal material from HT-Q. In contrast, IFNγ^+^ CD4 T cells were comparable between groups (Fig. 4*C*). As previously noted, we detected lymphocytic infiltrates in the ileum and colon of recipients of MS-Q but not in recipients of HT-Q-derived ileal microbiota (*SI Appendix*, Fig. S4*C*).

We profiled the fecal microbiota of all gnotobiotic mice 2 wk after colonization and the fecal and ileal microbiota at the endpoint. The colonization density was comparable in all animals averaging around 5 to 6 × 10^11^ bacteria/g of feces in both groups (*SI Appendix*, Fig. S4*D*) and recipient mice exhibited a similar alpha diversity in both groups (*SI Appendix*, Fig. S4*E*). While bacterial patterns remained remarkably stable over time in recipients of ileal microbiota from HT-Q (Fig. 4*D*), we noted highly dynamic changes in the mouse group receiving ileal material from MS-Q. Three out of 5 MS-Q-recipient mice that had developed EAE showed a relative abundance of about 75% of *Lachnoclostridium* (another member of the Lachnospiraceae family) at the endpoint of the experiment. In the remaining two diseased animals, this bacterium was also substantial in abundance, though to a lesser degree (38 to 47%), along with a major contribution of *Bacteroides* (45 to 55%). The same mixed pattern was seen in the three recipients of MS-Q-derived ileal material which remained healthy. Notably, *Bacteroides* were already of substantial abundance in MS-Q-recipient mice at the 2-wk time point, indicating that the putatively disease-associated expansion of *Lachnoclostridium* happened during the latter time interval. Interestingly, *E. tayi* was not detectable here. The differences between recipients of HT-Q and MS-Q-derived ileal microbiota are reflected in a significantly different beta diversity in both the ileum and feces of recipient mice at the experimental endpoint (*SI Appendix*, Fig. S4*F*). Further, similar to our initial observation (Fig. 3), other bacteria, like *Akkermansia, Alistipes,* and *Streptococcus,* which over time were substantial components in recipients of HT-Q ileal microbiota, were essentially lost in the MS-Q recipient group (Fig. 4*E* and *SI Appendix*, Fig. S4*G*).

### Expansion of Lachnospiraceae in Recipients of Ileal Material from Male MS Twin.

**Fig. 5.**
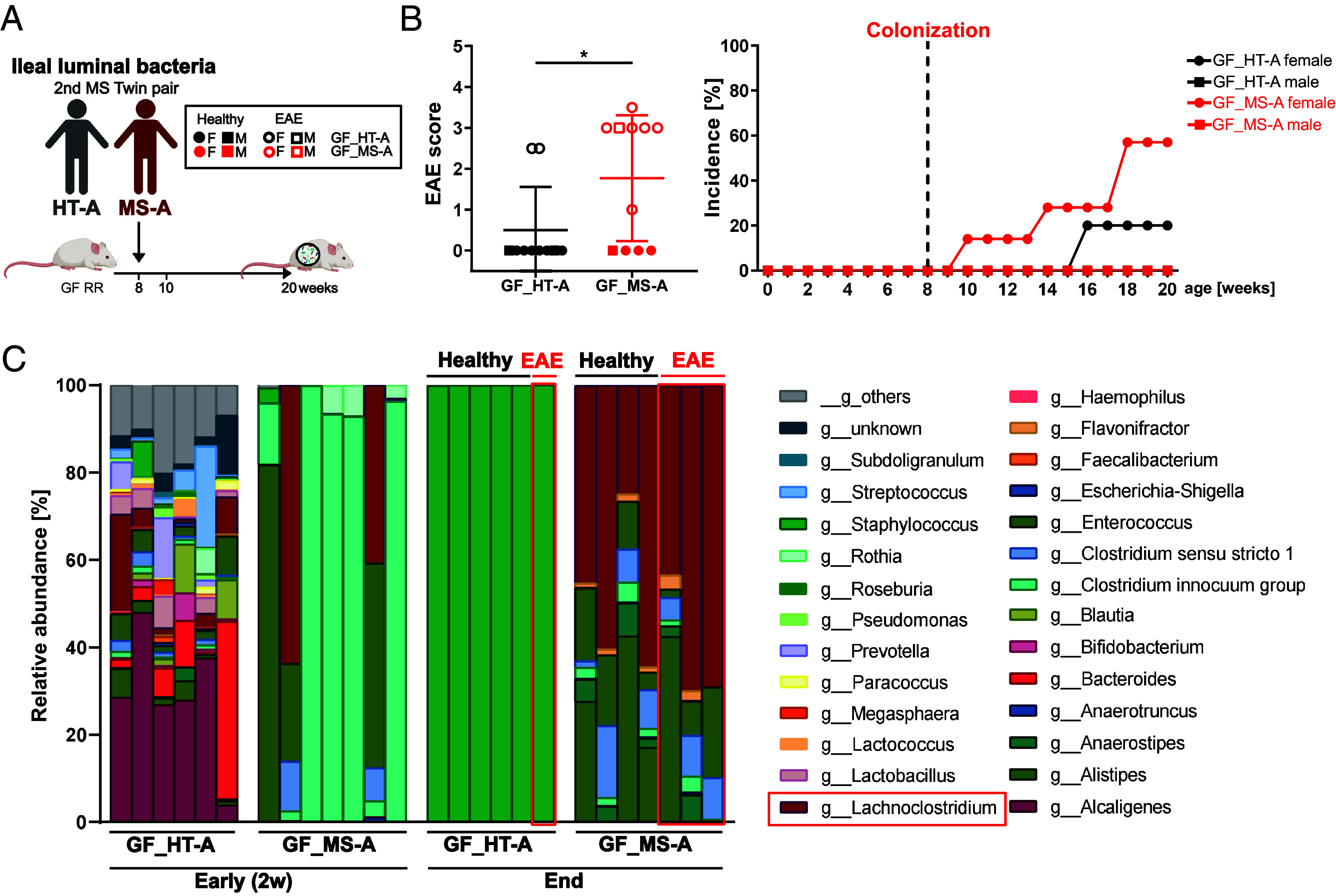
*Lachnoclostridium* is important for facilitating development of CNS inflammation. (*A*) Experimental scheme: Ileal bacteria from a second pair of twins were colonized into gnotobiotic RR SJL/J mice (GF_MS-A, n = 11 and GF_HT-A, n = 9). Created with BioRender. (*B*) EAE scores and incidence of EAE in the humanized gnotobiotic RR mice (unpaired *t*-test). (*C*) Relative abundance of different genera of the fecal microbiota from humanized gnotobiotic RR mice 2 wk after colonization (early) and at the endpoint. *Lachnoclostridium* was enriched in MS-A colonized RR mice at the endpoint and is framed in red.

To determine whether colonization of RR recipients with ileal material from a separate twin pair would result in similar or completely different microbial profiles and disease manifestation, we selected another twin pair, MS-A and HT-A, for colonization. While the previous donors were female, and the affected twin (MS-Q) had long disease duration with high disease activity and progression (EDSS 6.0), this second pair was male, and the affected twin (MS-A) was diagnosed with MS after a Clinically Isolated syndrome (CIS) with low disease activity and no progression under ongoing Interferon-beta treatment (EDSS of 1.5) at the time of enteroscopy. We colonized 11 GF RR mice (2 males and 9 females) with ileal material from MS-A, and 9 RR mice (2 males and 7 females) with ileal samples from HT-A (Fig. 5*A*). Notably, 6 out of 9 female RR recipients and 1 out of 2 male recipients developed EAE in the MS-A-colonized group, whereas only 2 out of 7 females and none of the two males developed EAE in the HT-A-colonized group (Fig. 5*B*). Although in this colonization experiment we observed EAE development also in two recipients of the HT-A group, recipients of MS-A material got sick at a significantly higher proportion, thus in line with our previous results.

Remarkably, profiling the fecal microbiota of recipient mice 2 wk after colonization and at the endpoint of the experiment again revealed an expansion of *Lachnoclostridium* in recipients of ileal microbiota from MS-A at the endpoint, which was not yet evident at the 2-wk time point prior to onset (Fig. 5*C* and *SI Appendix*, Fig. S5). However, this time the relative abundance of *Lachnoclostridium* was not different between sick and healthy mice. Of note, *E. tayi* was not detected in the recipient mice. Surprisingly, in recipients of ileal material from HT-A, we also detected expansion of a single species at the endpoint (i.e. *Staphylococcus*) (Fig. 5*C* and *SI Appendix*, Fig. S5), but this blooming was not associated with higher incidence of EAE. Taken together, our results show an association of members of the Lachnospiraceae family derived from ileal material with EAE development across two independent and highly heterogeneous MS twin pairs.

## Discussion

The risk of developing MS is tightly associated with changes of the intestinal microbial profile, as revealed by large population-based association studies (20). These ground-breaking investigations added an unexpected facet to our understanding of MS, but they were not designed to identify the mechanisms underlying this association, nor to functionally characterize or locate the microbial agents driving the pathogenesis. Here, we used a human-to-mouse transfer model to identify disease-related gut bacteria isolated from the small intestine of people with MS. This model allows unbiased scanning of the intestine for bacteria capable of sparking MS-like autoimmune disease in GF EAE-prone transgenic mice. Using this approach, we identify two recently characterized and related gut bacteria, *Eisenbergiella tayi* and *Lachnoclostridium* (both members of the Lachnospiraceae family), which qualify as disease-inducing agents.

Our search strategy rests on three pillars: It compares microbiota from genetically identical donors with or without MS; it examines gut microbiota from the small intestine; and it tests the autoimmune potential of gut bacteria in an in vivo model of human MS.

Identification of MS-associated microbes is hampered by the immense numbers of organisms and species contained within the intestinal microbiota, and its dynamic variability over time. This complexity is controlled both by the genetic and environmental diversity of human individuals (lifestyle, diets, etc.) (2). First-generation population-based association studies employed relatively small and not well-controlled cohorts that did not properly account for this high diversity and therefore yielded inconsistent results. However, newer studies such as the recent large study conducted by the *international MS Microbiome Study* reduced environmental variables by comparing MS-associated intestinal microbiomes of 1,100 home sharing participants (3). We further refined this approach here by additionally minimizing confounding genetic variability. To do so, we performed pairwise comparisons within a cohort of monozygotic twin pairs discordant for MS, who shared their parental home throughout adolescence (21, 22). While monozygotic twins share most if not all DNA encoded gene variants, including MS risk variants, they are by no means *identical* individuals. Thus, this strategy has proven invaluable for studies of MS-related changes in epigenetic DNA modification (22), and global immune signatures of T cell responses (12, 21, 23). Comparing fecal samples from 81 monozygotic twins discordant for MS, we identified 51 taxa to be differentially abundant. These included taxa that have previously been reported, such as *Anaerotruncus colihominis*, which was shown to be increased in MS patients in two other studies (9, 24), but which surprisingly ameliorates disease in an active-immunization EAE model (25). We also identified novel taxa, including, for example, an increase of *Copromonas*, *Acutalibacter,* and *Alistipes ihumii*, as well as a significant decrease, for example, in *Dialister succinatiphilus* and *Prevotella buccae*. Notably, both *D. succinatiphilus* and *P. buccae* are propionate producers, and although these exact species have to our knowledge not been detected in other studies, several reports describe reductions in short-chain-fatty-acid-producing classes of bacteria (3, 24). Furthermore, *E. tayi* was significantly increased in our cohort, as well as in the large household-controlled study (3), indicating that—as a low-abundant taxa—it might be detected only in well-controlled cohorts.

While most published studies relied only on fecal material (26), we additionally obtained samples from the terminal ileum and colon, the main sites of pathogenic interactions between microbiota and immune cells, by enteroscopy. Although due to the obligate cleansing procedure, a substantial fraction of the intestinal bacteria (mostly the planktonic and less so the mucus and wall-associated organisms) is lost and we thus cannot claim to cover the original composition of segment-specific microbiota, the remaining bacteria can be interrogated for their disease-inducing potential in our experimental system. This sampling strategy considers the diverse immune functions exerted in segmental gut milieus (27). While the small intestine is a site favorable for proimmune activities (28), anti-inflammatory processes seem to prevail in the large intestine. In particular, in the mouse, the small intestine harbors the most active interaction between bacteria and immune cells, notably including the activation of potentially CNS autoaggressive Th17 cells (29).

Classic association studies did not unequivocally determine the functional potential of disease-associated bacteria. For example, *Akkermansia muciniphila*, a mucus-degrading bacterium, was increased in MS in several reports, indicating a proinflammatory function (30). Paradoxically, however, in actively induced EAE, this organism ameliorated disease (9). Functional association between gut organisms and MS has been approached by transferring suspected organisms to actively induced rodent models (8). In contrast, here we transferred human-derived bacteria into a refined spontaneous EAE model, with a proven increased potential to develop MS-like autoimmune disease upon transfer of MS-derived fecal microbiota (7). In the present study, we applied this model for an unbiased search for pathogenic human ileum-derived bacteria.

The incidence of EAE was highest in recipients of ileal samples from twin donors with MS, although due to the small number of colonizations with ileal material, this requires confirmatory experiments in the future. Nonetheless, in all sick mice, the microbiota exhibited strong expansion of Lachnospiraceae, namely *E.tayi* and *Lachnoclostridium*. Notably, these expanded taxa, especially *E. tayi*, were minor components in the human specimens, although relatively increased in MS donors (3). Pathogenic activity of the dominant bacteria should be confirmed in future experiments by monocolonization of GF RR mice with cultured *E. tayi* and *Lachnoclostridium* organisms isolated from recipients of MS twin–derived ileal samples.

The striking association of dominant outgrowth (“blooming”) of *E. tayi* and *Lachnoclostridium* with the spontaneous development of EAE warrants particular attention. It seems that these organisms can autonomously facilitate EAE, without the support of ancillary bacterial species (31). Bacterial expansion may be fostered either by an especially supportive local milieu or by lack of suppression by competing bacteria that keep the Lachnospiraceae at low levels in the human donor. Conversely, the EAE-inducing species seems to be able to suppress competing donor-derived bacteria in the recipient intestine (32, 33). Triggering of EAE by bacterial expansions was restricted to *E. tayi* and *Lachnoclostridium*, while blooming of unrelated taxa (e.g., *Akkermansia*) failed to spark CNS autoimmunity. While still understudied, the compositional dynamics of bacterial communities in the human gut might be an important factor in the observed outcome (34).

Both *E. tayi* and *Lachnoclostridium* organisms belong to the Lachnospiraceae family of gut bacteria, a frequent component of the intestinal tract (35). They are obligate anaerobic spore-forming bacteria. However, the function of these organisms, in particular their interaction with the gut-associated lymphoid tissue (GALT), remains fragmentary and controversial. Thus, some Lachnospiraceae fueled inflammation in an inflammatory bowel disease (IBD) model by mobilizing proinflammatory macrophages (36), but in another study, similar bacteria mitigated IBD via the anti-inflammatory receptor NLRP12 (37). Alternatively, antigenic mimicry may play a role, similar to that reported for segmented filamentous bacteria (SFB) (38). Finally, it is known that the diet strongly modulates the gut microbiota composition and the transit time of digesta through the colon influences the activities of gut microbiota.

We noted that EAE incidence with bacterial expansion preferentially affected female recipient mice, an observation that recalls the well-known female preponderance in human MS (39). A marked sex dimorphism with enhanced susceptibility to EAE is also present in female SJL/J mice. Indeed, we reported that female RR mice preferentially develop a relapsing-remitting EAE course, while males tend to chronic disease (19). Furthermore, enhanced EAE activity in female mice has been observed in actively induced and T cell–mediated models of SJL/J mice (40). The mechanisms underlying the sex dimorphisms remain unclear. Transfer studies linked this diversity to sex dependent functional differences of effector T cells (41). There seems to be a role of genes on the sex chromosomes (42), and one study noted estrogen effects in C57BL/6 mice on microbiota before and following EAE induction (43). A causal link to gut microbiota has not been observed in CNS autoimmunity, although in NOD mouse diabetes, fecal transplantation from adult males to immature females suggested microbial effects on sex hormone production and regulation but did not assess the microbial profiles of the recipients (44).

Recent imaging studies documented the direct activation of CNS autoimmune CD4^+^ T cells within the small intestinal lamina propria and the subsequent migration of the activated T cells into the CNS target tissue. The intestinal activation requires the presence of local microbiota and involves MHC class II structures (45). It drives the T cells into a Th17-like differentiation path distinct from active subcutaneous immunization (46). The intestinal interactions instigate the originally innocuous self-reactive T cells to turn into pathogenic aggressors. Notably, in recipients of MS ileal material we observed phenotypic changes in the T cell response with increased IL-17 production, whereas IFNγ-producing Th1 cells were not significantly increased. One obvious activating mechanism would involve molecular mimicry, i.e. a cross-reactivity of T cells recognizing the autoantigen MOG and some bacterial structure (29, 38). Alternatively, the bacteria could bypass T cell receptor activation through a facilitating metabolite as reported in the case of SFB (47). In the latter case, the bacteria would not necessarily activate autoimmune T cells directly but could create a microenvironment that facilitates T cell activation indirectly. In addition to pathogenic activation of effector T cells, depletion or silencing of self-protective Tregs may occur. Indeed, directly comparing ileal material of MS versus healthy twins, recipients of MS samples exhibited a trend toward reduced Foxp3-positive cells. Irrespective of the exact pathogenic mechanisms, our studies point to the involvement of *E. tayi* and possibly also *Lachnoclostridium* in the pathogenic conversion process.

Mechanistic studies of microbial functions in human diseases are limited by technical and ethical constraints. One way to overcome these hurdles has been the transfer of microbiota from humans to GF mice, a strategy which has been successfully used to investigate the role of microbial dysfunctions in diverse clinical disorders including metabolic, but also immune related diseases (48). However, this strategy raises questions about the representative power of trans-species transfer strategies. Obviously, the immune systems differ between humans and mice (49). Further, the intestinal microenvironments differ between the species, which impacts microbial composition and metabolism. Furthermore, the species profoundly differ by their “lifestyle” (diet, etc.), which further impacts the microbial profiles, and, in consequence, changes the composition of human-derived microbiota substantially upon transfer to mice (50).

All these limitations aside, the two species share their fundamental organization (51). In particular, the transgenic MS model used here, the RR mice, spontaneously recapitulates many of the clinical and structural features of (early) human MS. Yet, there are differences at the same time. RR EAE is driven by CD4^+^ T cells recognizing MOG, an immunodominant target autoantigen in several mouse strains, while in MS the nature of target autoantigens is more complex. Recent work indicates a role of MOG-specific CD4^+^ T cells just in a group of about 20% of human MS patients (52). By contrast, anti-MOG autoantibodies are diagnostic for a separate entity of demyelinating disorder called MOGAD (53). Weighing pro- versus antiarguments, we consent with Arrieta et al. who conclude that “Human microbiota associated mice are currently the best (if not the only) model available to study the role of disease-associated microbiome alterations” (54).

### Outlook and Conclusion.

We present a unique experimental paradigm by first studying fecal microbiota on a large cohort of monozygotic twins discordant for MS, an approach that drastically reduces potential confounding factors. Second, we enteroscopically sampled ileal microbiota from selected twins and introduced them into GF transgenic mice. Our experimental strategy exhibits unique strengths but also limitations that need to be considered (Table 1). Our results to date implicate two members of the Lachnospiraceae family residing in the ileum as potential candidates for triggering clinical disease. For obvious reasons, our unbiased human-to-mouse transfer experiments could only be performed with a very small number of selected human twin donors. It was therefore important to assess the presence of the functionally incriminated bacteria also in fecal material from our large cohort of MS-discordant monozygotic twins (n = 81). Using pairwise comparison, we found a significant increase of *E. tayi* in the MS twins compared to their healthy twins. Together with our functional studies, this supports our conclusion that these bacteria might play a crucial role as environmental triggering factors of human MS, although further studies will be required to extend our present findings.

**Table 1.** Strengths and limitations of our study

Strengths	Limitations
•Comparison of MS-discordant monozygotic twin pairs reduces confounders to a minimum	•MS-discordant twin pairs volunteering to undergo enteroscopy are limited, thus reducing the number of colonization experiments
•Sampling from the ileum (instead of feces) focuses on microbiota interacting closely with the GALT	•Interspecies differences of physiology and immune responsiveness need to be considered
•Use of a transgenic mouse model carrying a MOG-specific TCR, that spontaneously develops EAE depending on intestinal microbiota	•Bowel cleansing procedure may lead to loss of microbial taxa •Human bacteria strains may differ in their vulnerability to storage conditions
•Transfer into GF model mice allows to correlate composition of human microbiota with the incidence of spontaneous EAE •Identification of EAE-triggering bacteria without biased design	•Spontaneous EAE develops over extended periods of time and may not always manifest within the approved experimental timeframes (loss of late-appearing disease cases)

Paradoxically, while two members of the Lachnospiraceae showed a strong encephalitogenic potential, these taxa represent minor components in the human fecal microbiome and thus, have escaped notice in most profiling studies. One attractive explanation to resolve this inconsistency involves formation of bacterial biofilms. Indeed, biofilms are formed by single or multiple organisms that colonize inner surfaces along the intestinal tube. These assemblies are embedded in a matrix at high density allowing intense and sustained interaction with the GALT, but by their overall contribution to the global intestinal microbiota might be too low to be recognized in the fecal population (55).

Our studies are still in a pilot and proof-of-concept phase, allowing the first direct identification and localization of MS-derived microbes capable of triggering MS-like disease in susceptible mice. However, these may not be the only organisms with this capability. Future studies applying similar strategies will provide a more complete picture. If indeed the MS-facilitating potential of the intestinal microbiota turns out to be restricted to a well-defined taxon, this may open up therapeutic opportunities. Noninvasive, selective modification of the microbiome would be a most valuable addition to the existent manipulation of the immune system.

## Materials and Methods

### Animals.

RR mice (TCR_1640_ mice) carry a transgenic TCR specific for the MOG_96-105_ peptide on the SJL/J background (19). All animals in this study were housed and bred in the germfree facility at ZIEL—Institute for Food and Health, Technical University of Munich, Freising, Germany. Animal experiments were designed and performed in accordance with regulations of the animal welfare acts and under approval by the animal ethics committee of the state of Bavaria (Regierung von Oberbayern) in accordance with European guidelines.

### MS Twin Cohort and Study Design.

The MS twin cohort used in this study was part of the MS TWIN STUDY, based at the Institute of Clinical Neuroimmunology at the University Hospital of the LMU Munich, Germany, where to date 101 monozygotic twin pairs discordant for MS are enrolled. Inclusion criteria for study participation were met only if one cotwin was affected by MS, whereas the cotwin was clinically healthy. Exclusion criteria were infection and antibiotic treatment or high-dose intravenous glucocorticosteroids within the three months before the visits that included biosampling. Twin pairs visited the outpatient department at the Institute of Clinical Neuroimmunology in Munich for a detailed interview, neurological examination, MRI, and sampling of biomaterials including blood, feces, and sometimes CSF. All participants completed surveys to report demographics, lifestyle and physiology factors, and dietary habits. To confirm MS diagnosis, medical records, including MRI scans, were obtained and reviewed.

For this study, frozen fecal samples of 81 twin pairs (see basic clinical characteristics in *SI Appendix*, Table S2) were processed using the QIAamp PowerFecal DNA kit for 16S rRNA sequencing in an automated Eppendorf epMotion5070 liquid handling apparatus. The V3 to V4 variable region of the bacterial 16S rRNA gene was amplified and sequenced on an Illumina Miseq system. 16S rRNA sequencing analysis and statistical analysis of fecal samples from MS twins (shown in Fig. 1) is described in *SI Appendix*.

Four twin pairs consented to enteroscopic sampling of intestinal microbiota from the terminal ileum and colon, along with fecal material. Enteroscopic sampling was performed after a standard cleansing procedure with sodium picosulfate solution during a routine ileocolonoscopy for colon cancer screening. Mucosal biopsies were obtained using sterile biopsy forceps (disposable EndoJaw, Olympus), (n = 2 to 4/site, size each approx. 2 mm diameter). Ileal and colonic fluid (10 mL/site) were collected into 15 mL tubes via a sterile catheter (V-system single-use cannula, Olympus). Biopsies were immediately conserved on dry ice. Intestinal bacteria from the lavage fluid were immediately isolated by centrifugation. Sample processing was performed under anaerobic conditions. Isolated bacteria and biopsy samples were preserved in 20% glycerol at −80 °C. 16S rRNA gene sequencing of enteroscopically obtained samples from MS twins (shown in Fig. 2) is described in *SI Appendix*.

The MS TWIN STUDY including all procedures, investigations, biobanking, and patient consent has been approved by the local ethics committee of the University of Munich, Germany, project certification number MS TWIN STUDY 267-13, MS TWIN STUDY with colonoscopy: Project 567-16. All patients gave their written informed consent.

### Colonization of Gnotobiotic Mice with Bacteria.

The bacterial supernatant was transferred to Hungate tubes under anaerobic conditions. Germfree RR mice were bred and housed in isolators and then transferred to Isocage P System (Tecniplast) cages ventilated via HEPA-filtered air for colonization. Mice received 100 µL of the ileal bacteria suspension (approx. 2 × 10^6^ bacterial cells) via oral gavage at 8 wk of age. Feces were collected 2 wk after colonization and at the endpoint. Recipients were killed 12 wk after colonization or after the first signs of paralysis occurred. Animals were monitored for clinical signs of EAE according to the following scoring criteria: 0, no disease; 1, animal with a flaccid tail; 2, animal with impaired righting reflex and/or gait; 3, animal with one paralyzed hind leg; 4, animal with both hind legs paralyzed; and 5, moribund animal or death of the animal after preceding clinical disease.

16S rRNA gene sequencing of fecal and intestinal contents of colonized mice, as well as histological analyses, cellular analyses by flow cytometry and detection of MOG-specific antibodies in colonized mice (Figs. 3–5) are described in *SI Appendix*.

### Statistical Analysis.

Statistical analysis was performed using GraphPad Prism 7. A two-tailed unpaired Student’s *t*-test was used for comparison of two groups including maximum EAE scores, anti-MOG IgG levels, bacterial counts in feces, flow cytometry data, and relative abundance of individual taxa. One-way ANOVA with Tukey’s multiple comparisons test was used for multiple comparisons including alpha diversity of different intestinal sites and relative abundance of individual taxa 2 wk after colonization versus endpoint. *P* values <0.05 were considered significant. *P < 0.05; **P < 0.01; ***P < 0.001.

## Supplementary Material

Appendix 01 (PDF)

## Data Availability

16S rRNA sequencing data from the colonization experiments in this study were deposited on NCBI Sequence Read Archive and are accessible under PRJNA1197428 (56).
