# Supplementary material for: Multiple sclerosis and gut microbiota: Lachnospiraceae from the ileum of MS twins trigger MS-like disease in germfree transgenic mice—An unbiased functional study
Source: Proc Natl Acad Sci U S A. 2025 Apr 21;122(18):e2419689122. doi: 10.1073/pnas.2419689122 (PMC12067282; doi:10.1073/pnas.2419689122)
Supplement: Supplementary file 1 — Appendix 01 (PDF) [file pnas.2419689122.sapp.pdf]

PNAS

Supporting Information for

Multiple Sclerosis and Gut Microbiota: *Lachnospiraceae* from the Ileum of MS Twins trigger MS-like Disease in Germfree Transgenic Mice

Hongsup Yoon[1,2,3§], Lisa Ann Gerdes[1,2,4§], Florian Beigel[5§], Yihui Sun[6], Janine Kövilein[1,2,3], Jiancheng Wang[1,2], Tanja Kuhlmann[7], Andrea Flierl-Hecht[1], Dirk Haller[8], Reinhard Hohlfeld[1], Sergio E. Baranzini[6#], Hartmut Wekerle[1,3*#] & Anneli Peters[1,2*#]

[1] Institute of Clinical Neuroimmunology, University Hospital Ludwig-Maximilians-Universität München, Munich, Germany

[2] Biomedical Center (BMC), Faculty of Medicine, Ludwig-Maximilians-Universität München, Martinsried, Germany

[3] Max Planck Institute for Biological Intelligence, Martinsried, Germany

[4] Munich Cluster of Systems Neurology (SyNergy), Munich, Germany

[5] Department of Medicine II, University Hospital, LMU Munich, Germany

[6] Weill Institute for Neurosciences, Department of Neurology, University of California San Francisco, San Francisco, CA, USA

[7] Institute of Neuropathology, University Hospital Münster, Münster, Germany

[8] ZIEL Institute for Food & Health, Technical University of Munich, Freising, Germany

[§] shared authorship

[#] contributed equally

* Correspondence to Hartmut Wekerle: hartmut.wekerle@bi.mpg.de or Anneli Peters: Anneli.Peters@med.uni-muenchen.de

This PDF file includes:

Figures S1 to S5
Table S1 and S2
Extended material and methods
SI References

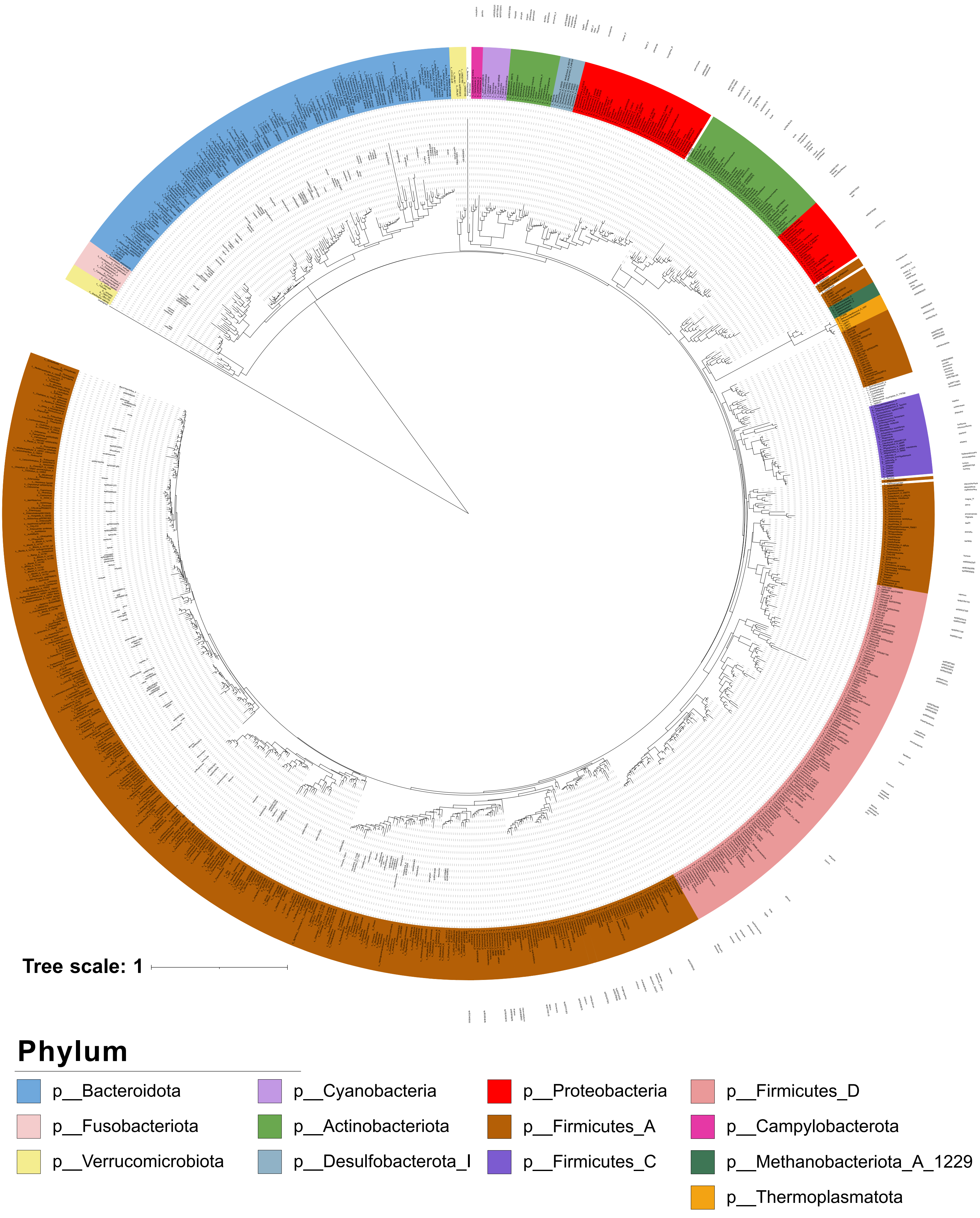

Fig. S1. Phylogenetic tree of all taxa identified by 16S rRNA gene sequencing.
The phylogenetic tree of all identified taxa is shown with each identified ASV colored by Phylum.

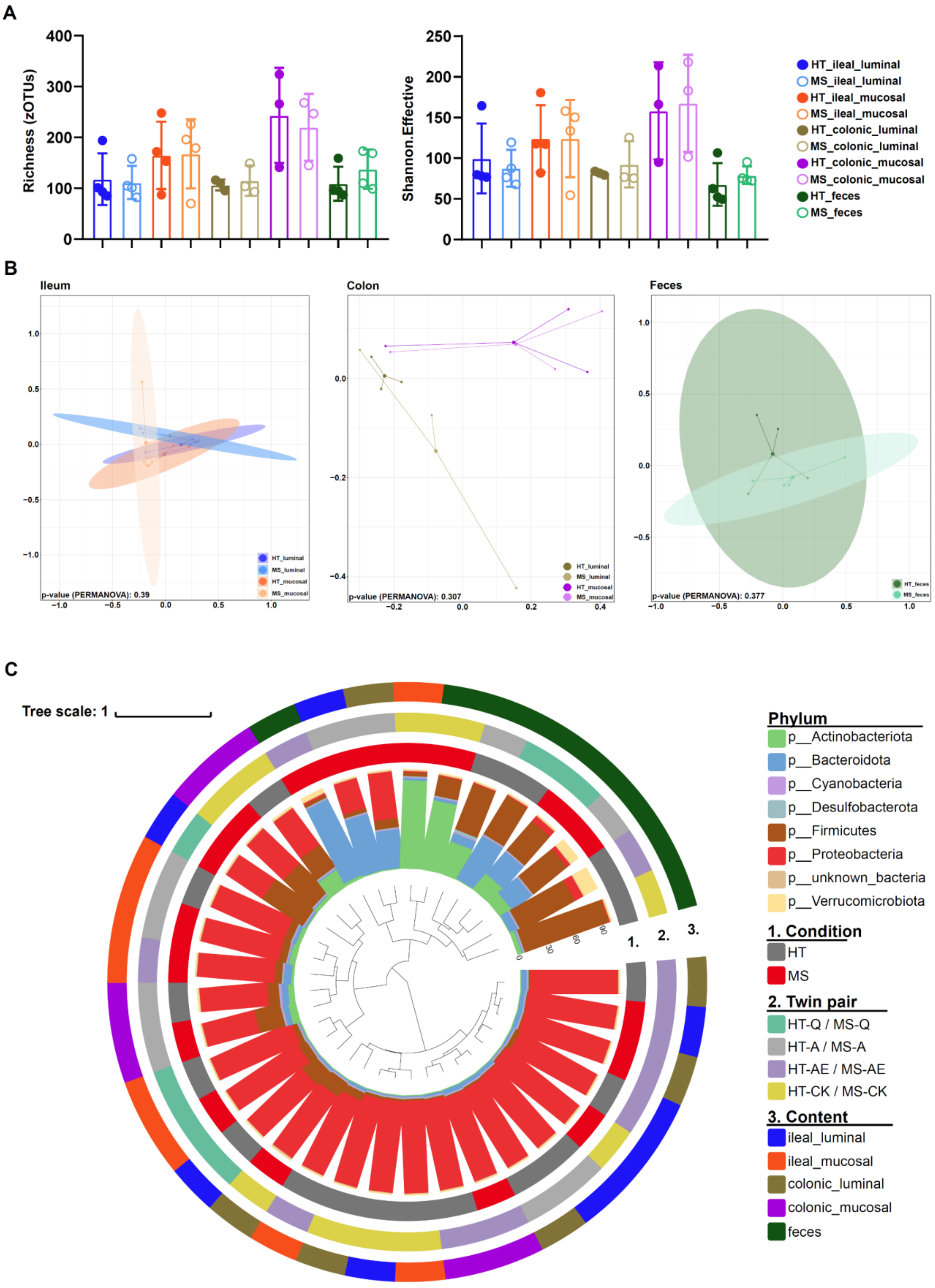

A
Richness (zOTUs)
Shannon.Effective
HT_ileal_luminal
MS_ileal_luminal
HT_ileal_mucosal
MS_ileal_mucosal
HT_colonic_luminal
MS_colonic_luminal
HT_colonic_mucosal
MS_colonic_mucosal
HT_feces
MS_feces
B
Ileum
Colon
Feces
p-value (PERMANOVA): 0.39
p-value (PERMANOVA): 0.307
p-value (PERMANOVA): 0.377
HT_luminal
MS_luminal
HT_mucosal
MS_mucosal
HT_feces
MS_feces
C
Tree scale: 1
Phylum
p__Actinobacteriota
p__Bacteroidota
p__Cyanobacteria
p__Desulfobacterota
p__Firmicutes
p__Proteobacteria
p__unknown_bacteria
p__Verrucomicrobiota
1. Condition
HT
MS
2. Twin pair
HT-Q / MS-Q
HT-A / MS-A
HT-AE / MS-AE
HT-CK / MS-CK
3. Content
ileal_luminal
ileal_mucosal
colonic_luminal
colonic_mucosal
feces

Fig. S2. Comparison of enteroscopically obtained microbiota from different intestinal sites from MS twins.

(A) Alpha diversity (richness and Shannon Effective) of the location-specific microbiota in MS twins, n=3 or 4 per group ($p > 0.05$, one-way ANOVA with Tukey's multiple comparisons test). **(B)** Beta diversity illustrated by PCoA plots of location-specific microbiota based on generalized UniFrac distance. Differences between groups were tested using PERMANOVA and were not significant ($p > 0.31$). **(C)** Dendrogram of location-specific microbiota profiles based on generalized UniFrac distances in MS twins. Individual taxonomic composition at phylum level is shown as stacked bar plots around the dendrogram. The first ring indicated the twins condition (HT or MS), followed by twin pairs in the second ring, and intestinal sampling sites of microbiota profiles (content) in the third ring.

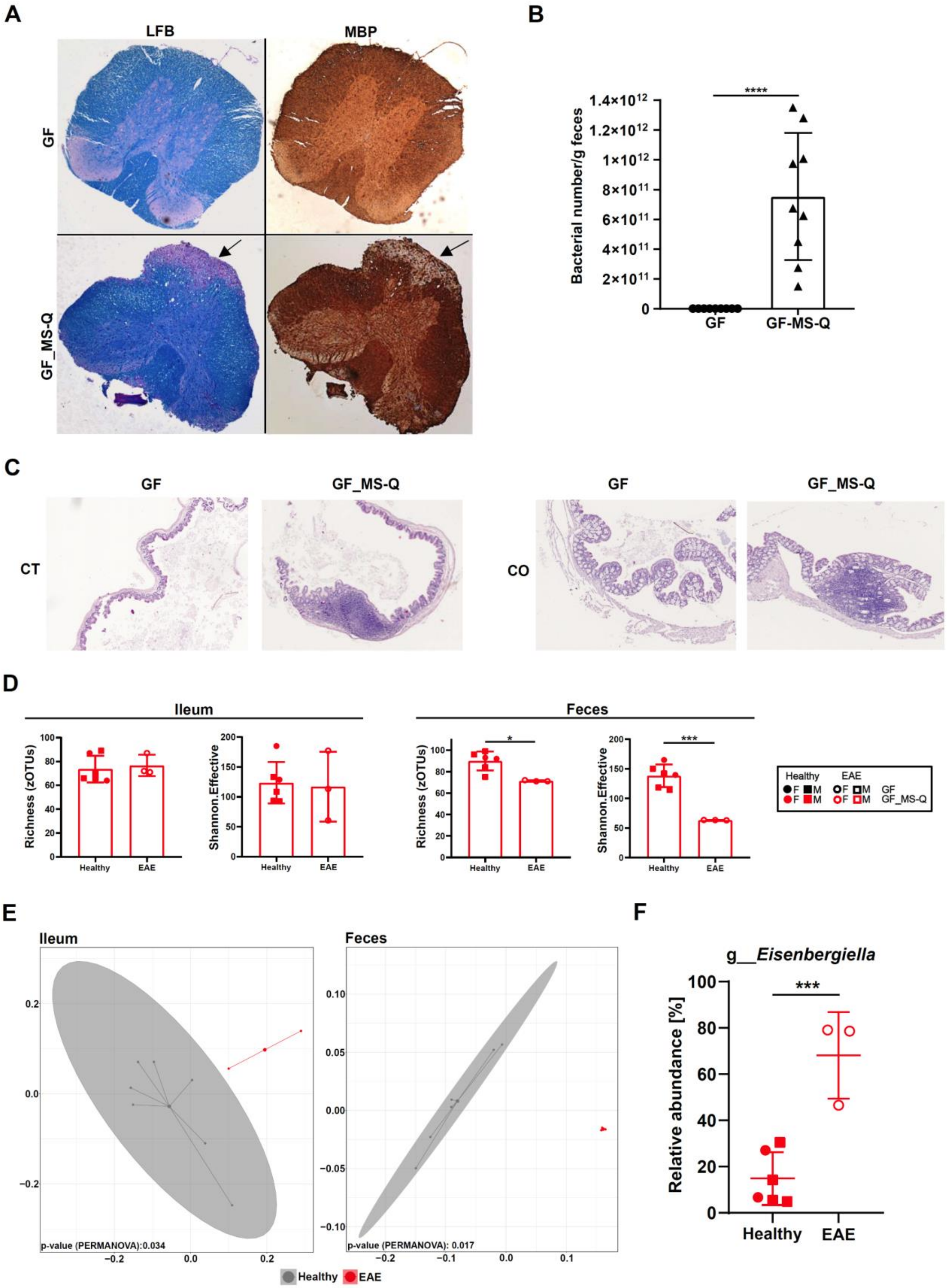

A
LFB
MBP
GF
GF_MS-Q
B
****
Bacterial number/g feces
GF
GF-MS-Q
C
GF
GF_MS-Q
CT
CO
D
Ileum
Feces
Richness (zOTUs)
Shannon.Effective
Healthy
EAE
*
***
Healthy EAE
F M F M GF
F M F M GF_MS-Q
E
Ileum
Feces
p-value (PERMANOVA):0.034
p-value (PERMANOVA): 0.017
Healthy
EAE
F
g__Eisenbergiella
***
Relative abundance [%]
Healthy
EAE

Fig. S3. EAE development and microbial features in humanized gnotobiotic RR mice.
(A) Representative pictures of demyelination (LFB staining and MBP staining) in the CNS of MS-Q colonized mice compared to non-colonized GF mice. **(B)** Number of bacteria in the feces of RR recipient mice (unpaired *t*-test). **(C)** Representative pictures of immune cell infiltration (H&E staining) in the cecum (CT) and colon (CO). **(D)** Alpha diversity (richness and Shannon Effective) of ileal ($p > 0.70$, unpaired t-test) and fecal ($p < 0.01$, unpaired t-test) microbiota. **(E)** Beta diversity illustrated by PCoA plots of ileal and fecal microbiota based on generalized UniFrac distances. Significances were tested using PERMANOVA ($p = 0.034$ for ileum, and $p = 0.017$ for feces). **(F)** Relative abundance of *Eisenbergiella* as sole genus of the ileal microbiota that showed a significant difference (unpaired t-test) between healthy and diseased animals colonized with MS-Q derived ileal microbiota.

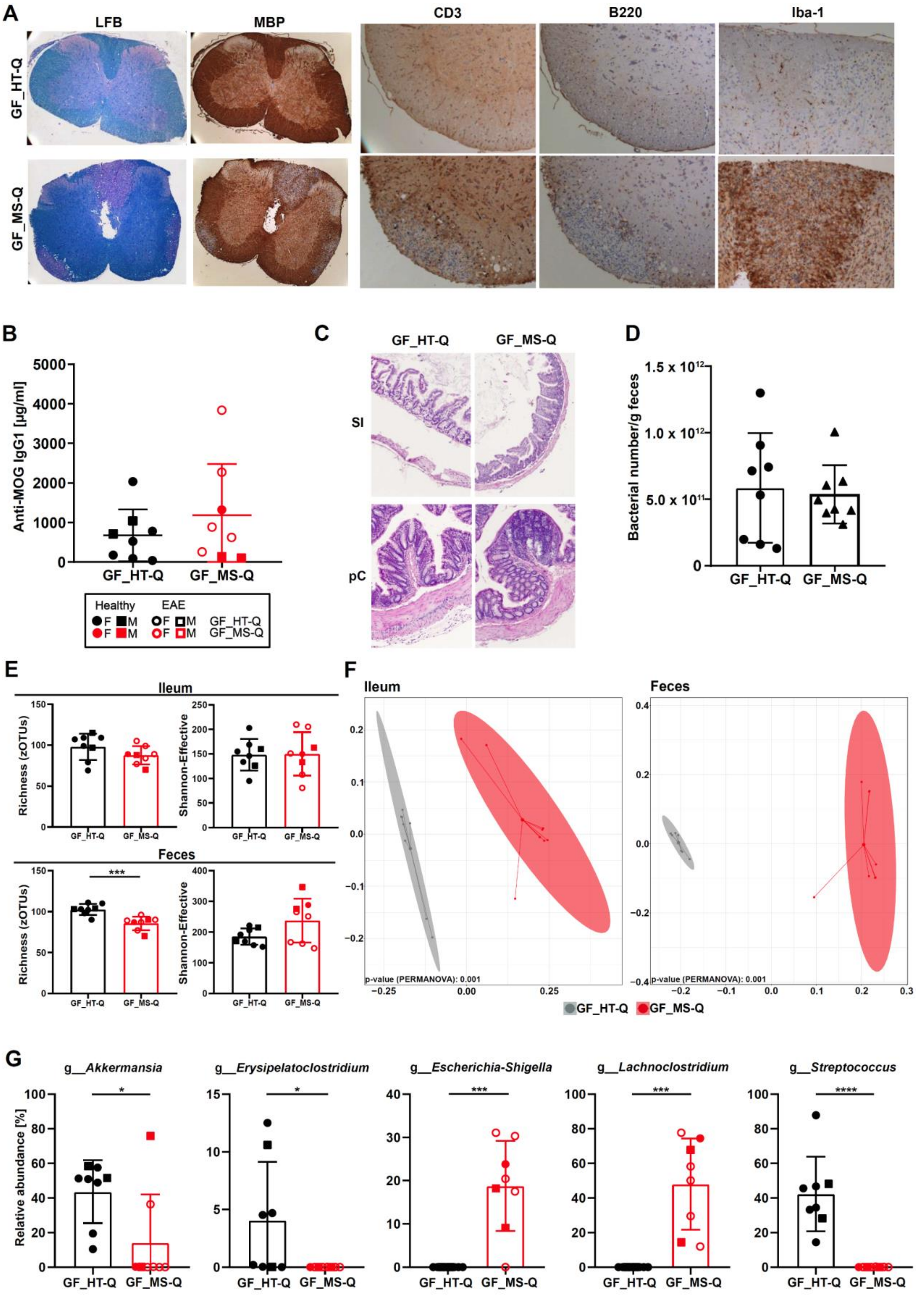

A
LFB
MBP
CD3
B220
Iba-1
GF_HT-Q
GF_MS-Q
B
Anti-MOG IgG1 [µg/ml]
GF_HT-Q
GF_MS-Q
Healthy
EAE
F
M
C
SI
pC
D
Bacterial number/g feces
GF_HT-Q
GF_MS-Q
E
Ileum
Feces
Richness (zOTUs)
Shannon-Effective
***
F
Ileum
Feces
p-value (PERMANOVA): 0.001
GF_HT-Q
GF_MS-Q
G
g__Akkermansia
g__Erysipelatoclostridium
g__Escherichia-Shigella
g__Lachnoclostridium
g__Streptococcus
Relative abundance [%]
*
***
****

Fig. S4. EAE development and microbial features in RR mice colonized with MS twin-derived vs. healthy twin-derived ileal material.

(A) Representative pictures of LFB and MBP staining in the CNS of colonized RR mice show demyelination in MS-Q recipients. Sections show modest infiltration of T cells (CD3) and B cells (B220) and strong signal for activated macrophages/microglia (Iba-1). **(B)** Anti-MOG ELISA in the serum of humanized gnotobiotic RR mice ($p > 0.34$, unpaired t-test). **(C)** Representative pictures of H&E staining of ileum (SI) and proximal colon (pC) of humanized gnotobiotic RR mice. **(D)** Number of bacteria in the feces of colonized RR mice ($p > 0.77$, unpaired t-test). **(E)** Alpha diversity (richness and Shannon Effective) of ileal ($p > 0.16$) and fecal ($p = 0.0005$ and 0.07, unpaired t-test) microbiota from GF RR mice colonized with ileal bacteria of healthy co-twin or MS twin at the time of sacrifice. **(F)** Beta diversity illustrated by PCoA plots of ileal and fecal microbiota of humanized gnotobiotic RR mice based on generalized UniFrac distances. Differences between groups were tested using PERMANOVA ($p = 0.001$). **(G)** Relative abundance of ileal microbiota in humanized gnotobiotic RR mice that showed significant differences (unpaired t-test) at the genus level.

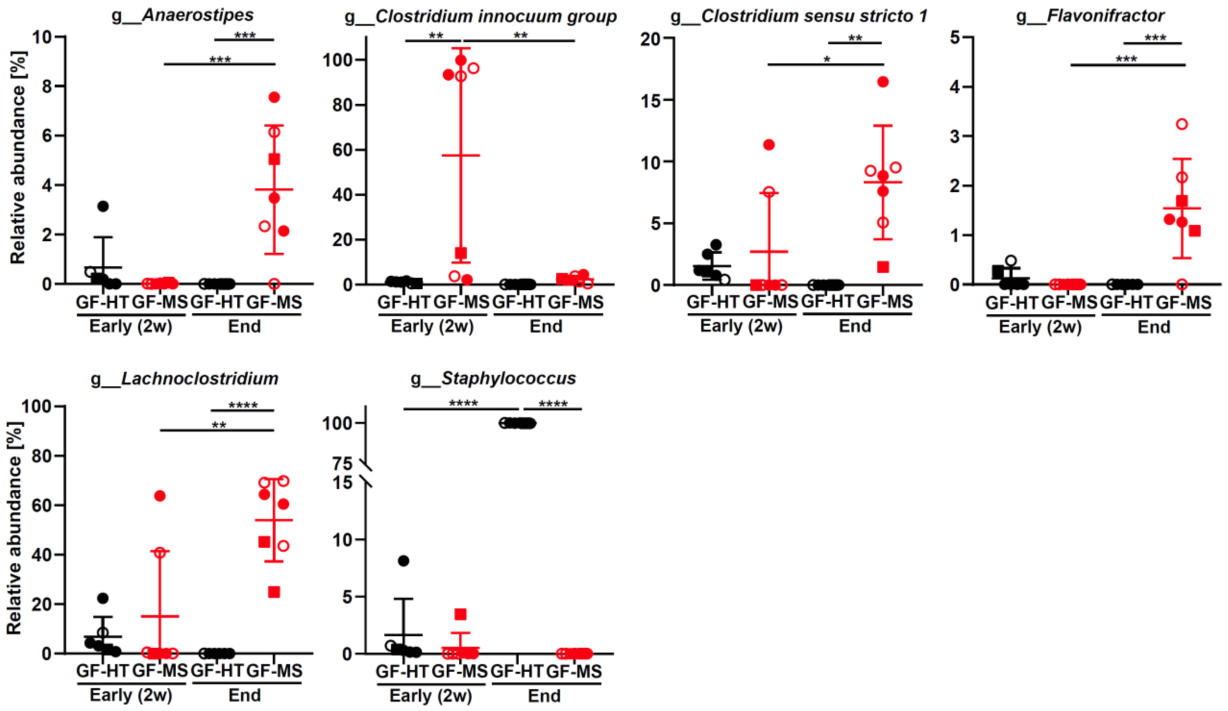

Fig. S5. Microbial features in RR mice colonized with MS-A / HT-A-derived ileal material. Relative abundance of fecal microbiota from humanized gnotobiotic RR mice that showed significant changes (unpaired t-test) at the genus level 2 weeks after the colonization and at the endpoint.

***Table S1**: Clinical characteristics of MS TWINS that underwent enteroscopy*

**ID**	**MS-A**	**HT-A**	**MS-Q**	**HT-Q**	**MS-AE**	**HT-AE**	**MS-CK**	**HT-CK**
**sex**	m		f		f		f	
**age**	35,2		58,2		44,7		42	
**BMI**	23	22	19	21	22	25	26	32
**disease course**	RRMS	healthy	SPMS	healthy	RRMS	healthy	RRMS	healthy
**disease duration [years]**	8,85	-	22,93	-	14,01	-	0,73	-
**EDSS**	1,5	-	6	-	1,5	-	2,5	-
**DMT**	IFN	-	none	-	DMF	-	FTY	-
**smoking**	smoker, 1pys	smoker, 1pys	smoker, 32 pys	smoker, 16 pys	never-smoker	never-smoker	never-smoker	never-smoker
**concomitant diseases**	none	none	none	none	none	fructose intolerance	vitiligo	vitiligo
**co-medication**	vitamine D, ibuprofen intermitt.	none	fluoxetin, pregabalin, baclofen, distigmin	none	vitamine D, thyroxine, ibuprofen intermitt.	Lactobacillus plantarum CECT 7315 & 7316 20 2 months prior colonoscopy	vitamine D, citalopram, hormonal contraception	citalopram, hormonal contraception, ibuprofen intermitt.
**diet**	omnivore diet, more milk	omnivore diet, more sweets, dietary supplement	omnivore diet, more milk, little meat	omnivore diet, less milk, more meat	omnivore diet, less dairy and milk	omnivore diet, more whole grain, fresh fruit and vegetables	omnivore diet, more fuits and vegetables, cooks daily	omnivore diet, more softdrinks, never cooks
**residence**	Germany, south east	Germany, south east	Germany, mid west	Germany, mid west	Germany, north east	Germany, mid west	Germany, south west	Germany, south west
**shared household**	until age 20	until age 20	until age 20	until age 20	until age 19	until age 19	until age 27	until age 27
**physical activity**	moderate	none	none	very active	moderate	moderate	none	none

Table S2: Clinical characteristics of MS twin cohort

Twin pairs	81
Age (y)	41.09 [21 -70]
Female	61
Disease course	
CIS	5
RRMS	61
SPMS	14
PPMS	1
EDSS	2.71 [0 - 9.5]
Disease duration EM (y)	12.44 [0.5 - 45.4]
Untreated	31
Treated	50
IFN	15
GLAT	8
FTY	8
NAT	6
DMF	5
LEM	2
OCR	2
TFN	2
CLAD	1
AZA	1

CIS = clinical isolated syndrome, RRMS = relapsing remitting MS, SPMS = secondary progressive MS, PPMS = primary progressive MS, EDSS = expanded disability status scale, IFN = interferon beta, GLAT = Glatirameracetate, FTY = Fingolimode, NAT = Natalizumab, DMF = Dimethylfumarate, LEM = Alemtuzumab, OCR = Ocrelizumab, TFN = Teriflunomide, CLAD = Cladribine, AZA = Azathioprin

Extended material and methods

<u>16S rRNA sequencing analysis and statistical analysis of fecal samples from MS twins (Fig. 1)</u>

The V3-V4 variable region of the bacterial 16S rRNA gene was amplified and sequenced on an Illumina Miseq system. The analysis for amplicon reads was performed with QIIME2 (1) (https://qiime2.org), trimming off bases with low scores was done with DADA2 (2) plugin and remaining reads were assigned to OTUs against Greengenes V4 taxonomy classifier (3). Samples that had very few reads (< 1000) were filtered out to minimize their impact on downstream statistical analysis.

Microbial composition was normalized as relative abundance and further log transformed. Species features with low variance (< $6.95e^{-12}$, corresponding to the lowest 10th percentile) were removed in order to reduce the impact of zero-inflation in microbiome data. A mixed linear regression model was applied on the transformed and filtered abundance data to identify significantly differential taxa between affected and unaffected twins with a fixed effect of health status and a random effect of twinsID. The *lmer* function from R package "lmerTest" was used to perform the mixed linear model. Given the limited power of this analysis, a Chi-squared test was also employed to identify significant species that exhibited differing trend (increasing or decreasing) between twins (p-value of <0.05 were considered significant).

Alpha diversity was measured by Shannon index (4). The Mann-Whitney U test was used to test the significance of the differences in alpha diversity between MS and healthy groups. Weighted UniFrac (5) distances were calculated among all samples, and principal coordinates analysis (PCoA) was utilized for visualizing beta diversity. Diversity analysis was performed on QIIME2. The impact of disease status on microbiome abundance variation was assessed using the PERMANOVA test (6) using the *adonis* function from the R package vegan (7), testing on weighted UniFrac distances of MS and healthy samples. In addition, specifying strata as "twinsID" aimed to account for within-twin pair variability. Empirical p-values were obtained via 999 permutations.

MAFFT (8) alignment was performed to make multiple sequence alignment for representative sequences for each OTU cluster and fasttree (9) was employed to infer phylogenetic trees with alignments of representative sequences. All these procedures were executed using QIIME2. The visualization of phylogenetic tree was using iTOL (10).

<u>16S rRNA gene sequencing of enteroscopically obtained samples from MS twins (shown in Fig. 2) and from feces of colonized mice (Fig. 3-5)</u>

Bacterial genomic DNA was extracted from frozen mucosal biopsies, intestinal fluids and feces of those twin pairs that underwent enteroscopy, as well as from gut contents of mice following a modified version of the protocol by Godon et al. (11, 12). Briefly, samples were transferred to 2ml screw cap microcentrifuge tubes containing 500mg sterile silica beads (0.1mm diameter; BioSpec Products) and 600µl DNA stabilizing solution (Stratec Biomedical), 250µl of 4 M guanidine thiocyanate (0.1 M Tris; pH 7.5) and 500µl of 5% N-lauroyl sarcosine (0.1 M phosphate buffer; pH 8.0) were added. Samples were incubated for 1h at 70°C while shaking (700 rpm). Afterwards, bacterial cells were mechanically lysed by bead beating using FastPrep® instrument (MP Biomedicals, three times for 40s, 6.5 m/s), followed by the addition of 15mg Polyvinylpolypyrrolidone (Sigma Aldrich) and

vortexing. After centrifugation for 3 min at 15 000 xg and 4°C, the supernatant was obtained and centrifuged a second time. Afterwards, 500µl of clear supernatant was transferred into a new tube, mixed with 50µg of RNase and incubated for 30 min at 37°C while shaking. Purification of genomic DNA was performed following the NucleoSpin® gDNA Clean-up Kit (Macherey Nagel) according to manufacturer's instructions. Concentrations of extracted DNA was determined using the NanoDrop® Spectrophotometer ND-1000 (ThermoFisher Scientific).

Library preparation, sequencing and data analysis were performed as described in Lagkouvardos et al. (13). Briefly, bacterial 16S rRNA genes containing V3-V4 variable region were amplified by PCR (25 cycles) using primers 341F-785R. After purifying the amplicons using the AMPure XP system (Beckmann), samples were pooled and sequenced in a paired-end modus (PE275) using a MiSeq system (Illumina) following the manufacturer's protocol and the PhiX standard library (25% (v/v)). Raw reads were processed using the IMNGS pipeline (14) based on the UPARSE approach (15). After demultiplexing, sequences were trimmed to the first base with a quality score <3 and then paired. Sequences with <300 and >600 nucleotides and paired reads with an expected error >3 were excluded from the analysis. The remaining reads were trimmed by 5 nucleotides on each end to avoid GC bias and non-random base composition. UCHIME was used to test the presence of chimeras (16). Zero-radius operational taxonomic units (zOTUs) were clustered at 97% sequence similarity. Taxonomies were assigned using the RDP classifier (17) at 80% confidence level and compared to the SILVA database project (18). Downstream analyses were performed using the R-package Rhea (https://github.com/Lagkouvardos/Rhea) (19). In brief, rarefaction curves were used to evaluate sequencing depth and eliminate low quality reads. After normalization of zOTU counts the percentage relative abundance was calculated. Alpha diversity was assessed based on species richness and Shannon effective diversity. Beta diversity analysis was calculated based on generalized UniFrac distances. Phylogenetic tree was visualized with the online tool EvolView (20).

<u>Histological analysis</u>

Terminal ileum and distal colon were fixed in 4% paraformaldehyde for 24h and embedded in paraffin (McCormick; Leica EG1150C). 5µm-thick sections were prepared. The sections from the intestine were stained with Hematoxylin and Eosin (H&E) using a multistainer machine (Leica ST5020). The inflamed area was visualized by using the M8 microscope and scanner (PreciPoint GmbH).

Spinal cords and brains were removed, fixed in 4% PFA and embedded in paraffin. Paraffin sections (4µm thick) were stained for H&E and Luxol Fast Blue-Periodic Acid Schiff (LFB-PAS). For LFB-PAS, sections were stained with 1% LFB solution for 72h, washed with 0.05% Li_2CO_3 and subsequently stained with 1% periodic acid followed by incubation with Schiff reagent. Afterwards, sections were washed in water, dehydrated and mounted. Immunohistochemistry was performed using a biotin-streptavidin peroxidase technique (DCS) and an automated immunostainer (AutostainerLink 48, Dako). Sections were pretreated with citrate/TE buffer (pH 6 or 9, Dako) in a steamer. The primary antibodies were specific to MBP (Abcam, ab7349, 1:4000), Iba-1 (Wako, 019-19741, 1:1000), B220 (BD Pharmingen, 550286, 1:200), and CD3 (Serotec, MCA

1477, 1:100). 3,3′-diaminobenzidine (Dako) was used as a color substrate and sections were mounted with Eukitt® mounting medium (O. Kindler GmbH) after dehydration.

Cell isolation and Flow cytometry

Single-cell suspensions from spleen were prepared by homogenization through a 70μm cell strainer (Thermo Fisher Scientific). To lyse the red blood cells, single-cell suspensions were incubated with lysis buffer (BioLegend). For surface staining, the cells were staining with PE-Cy7-conjugated anti-CD4 (RM4-5), BV605-conjugated anti-CD3 (145-2c11) antibodies after Fc blocking (anti-CD16/32, BD Biosciences). For intracellular staining, cells were fixed and permeabilized using the Transcription Factor Staining Buffer Set (eBioscience) and followed by staining with FITC-conjugated anti–IFN-γ (XMG1.2), PE-conjugated anti-IL10 (JES5-16E3), PerCP-Cy5.5–conjugated anti-IL-17 (eBio17B7), APC-conjugated anti-Foxp3 (FJK-16s). Used antibodies were purchased from eBioscience and Biolegend. All data were acquired on a FACS fortessa cytometer (BD Biosciences) and analyzed by using FlowJo software (TreeStar).

Detection of MOG-specific antibodies

For the quantification of serum titers of anti-MOG-specific antibodies, sera were collected from non-colonized and colonized GF RR mice. 10μg/ml rMOG was coated on a protein-binding ELISA plate (Nunc Maxisorp). After blocking, diluted serum (1:2000) was added to the plates and incubated at 4°C over night. After plate washing, bound IgG1 was determined using a biotin-conjugated anti-mouse IgG1 antibody (clone A-85-1) followed by Avidin conjugated Horseradish Peroxidase (BD Biosciences). After adding TMB substrate and stop solution (Biolegend) plates were analyzed at 450 nm.

SI References

1. Caporaso JG*, et al.* (2010) QIIME allows analysis of high-throughput community sequencing data. *Nat Methods* 7(5):335-336.
2. Callahan BJ*, et al.* (2016) DADA2: High-resolution sample inference from Illumina amplicon data. *Nat Methods* 13(7):581-583.
3. McDonald D*, et al.* (2023) Greengenes2 unifies microbial data in a single reference tree. *Nat Biotechnol*.
4. Shannon CE (1997) The mathematical theory of communication. 1963. *MD Comput* 14(4):306-317.
5. Lozupone C & Knight R (2005) UniFrac: a new phylogenetic method for comparing microbial communities. *Appl Environ Microbiol* 71(12):8228-8235.
6. McArdle BH & Anderson MJ (2001) FITTING MULTIVARIATE MODELS TO COMMUNITY DATA: A COMMENT ON DISTANCE-BASED REDUNDANCY ANALYSIS. 82(1):290-297.
7. Zapala MA & Schork NJ (2006) Multivariate regression analysis of distance matrices for testing associations between gene expression patterns and related variables. *Proceedings of the National Academy of Sciences of the United States of America* 103(51):19430-19435.

8. Katoh K & Standley DM (2013) MAFFT multiple sequence alignment software version 7: improvements in performance and usability. *Mol Biol Evol* 30(4):772-780.
9. Price MN, Dehal PS, & Arkin AP (2009) FastTree: computing large minimum evolution trees with profiles instead of a distance matrix. *Mol Biol Evol* 26(7):1641-1650.
10. Letunic I & Bork P (2021) Interactive Tree Of Life (iTOL) v5: an online tool for phylogenetic tree display and annotation. *Nucleic Acids Research* 49(W1):W293-W296.
11. Godon JJ, Zumstein E, Dabert P, Habouzit F, & Moletta R (1997) Molecular microbial diversity of an anaerobic digestor as determined by small-subunit rDNA sequence analysis. *Appl Environ Microbiol* 63(7):2802-2813.
12. Metwaly A*, et al.* (2020) Integrated microbiota and metabolite profiles link Crohn's disease to sulfur metabolism. *Nat Commun* 11(1):4322.
13. Lagkouvardos I*, et al.* (2015) Gut metabolites and bacterial community networks during a pilot intervention study with flaxseeds in healthy adult men. *Mol Nutr Food Res* 59(8):1614-1628.
14. Lagkouvardos I*, et al.* (2016) IMNGS: A comprehensive open resource of processed 16S rRNA microbial profiles for ecology and diversity studies. *Scientific reports* 6:33721.
15. Edgar RC (2013) UPARSE: highly accurate OTU sequences from microbial amplicon reads. *Nat Methods* 10(10):996-998.
16. Edgar RC, Haas BJ, Clemente JC, Quince C, & Knight R (2011) UCHIME improves sensitivity and speed of chimera detection. *Bioinformatics* 27(16):2194-2200.
17. Wang Q, Garrity GM, Tiedje JM, & Cole JR (2007) Naive Bayesian classifier for rapid assignment of rRNA sequences into the new bacterial taxonomy. *Appl Environ Microbiol* 73(16):5261-5267.
18. Quast C*, et al.* (2013) The SILVA ribosomal RNA gene database project: improved data processing and web-based tools. *Nucleic Acids Res* 41(Database issue):D590-596.
19. Lagkouvardos I, Fischer S, Kumar N, & Clavel T (2017) Rhea: a transparent and modular R pipeline for microbial profiling based on 16S rRNA gene amplicons. *PeerJ* 5:e2836.
20. Subramanian B, Gao S, Lercher MJ, Hu S, & Chen WH (2019) Evolview v3: a webserver for visualization, annotation, and management of phylogenetic trees. *Nucleic Acids Res* 47(W1):W270-W275.
